# Socio-economic position as an intervention against overweight and obesity in children: a systematic review and meta-analysis

**DOI:** 10.1038/srep11354

**Published:** 2015-06-26

**Authors:** Shunquan Wu, Yingying Ding, Fuquan Wu, Ruisheng Li, Yan Hu, Jun Hou, Panyong Mao

**Affiliations:** 1 Research and Technology Service Center, 302 Hospital of PLA, Beijing, China; 2 Department of Medical Microbiology and Parasitology, Second Military Medical University, Shanghai, China; 3 International Cooperation Laboratory on Signal Transduction, Eastern Hepatobiliary Surgery Institute, Second Military Medical University, Shanghai, China

## Abstract

Studies that investigated the association between socio-economic position (SEP) and obesity in children suggest inconsistent results. The aim of this study is to summarize and quantify the current evidence on SEP and risks of overweight and obesity in children aged 0–15 years. Relevant studies published between 1990 to Sep 4, 2014 were searched in Medline, Web of Science, Embase, and the Cochrane Database of Systematic Reviews. Risk estimates from individual studies were pooled using random-effects models, according to lowest vs the highest SEP category. A total of 62 articles were included in the meta-analysis. The odds of both overweight risk and obesity risk were higher in the children with lowest SEP than in those with highest SEP (OR, 1.10, 95% CI: 1.03–1.17, and OR, 1.41, 95% CI: 1.29–1.55, respectively). Sub-group analyses showed that the inverse relationships between SEP and childhood overweight and obesity were only found in high-income countries and in more economic developed areas. In conclusion, our study suggests that children with lower SEP had higher risks of overweight and obesity, and the increased risks were independent of the income levels of countries.

The prevalence of childhood overweight and obesity has been increasing at an alarming rate throughout the world. A previous study estimated that 23.8% of boys and 22.6% of girls in developed countries and 12.9% of boys and 13.4% of girls in developing countries were overweight or obese in 2013^1^. Overweight and obesity in early life are associated with increased risk of hypertension, heart disease, diabetes mellitus, and sleep disturbances in adulthood^2^,^3^. Many factors have been identified to explain the current dramatic global increase in the prevalence of overweight and obesity in children, which include: lack of physical activity, large birth weight, increased television viewing, parental obesity, maternal tobacco smoking during pregnancy, nutritional factors, genetic influence and more^4^,^5^,^6^,^7^.

There is also a growing body of evidence that suggests that socio-economic position (SEP) is a risk factor for obesity. Family income, living space, parental educational level and car or house ownership were reported to modify children’s behavior relative to energy balance, thus affecting the likelihood of childhood obesity^8^,^9^. However, this relationship was inconsistent in different studies. In some studies, lower level of SEP was an independent risk factor of overweight and obesity in children^10^,^11^,^12^, while in other studies, children with higher level of SEP were significantly more likely to be overweight or obese^13^,^14^,^15^.

For the prevention of childhood overweight and obesity, it is crucial to investigate how the diseases are patterned by SEP. To our knowledge, no quantitative review has been undertaken previously to summarize the relationship between SEP and childhood overweight and obesity. We therefore conducted a systematic review and meta-analysis of the evidence for the relation between SEP and risks of overweight and obesity in children aged 0–15 years.

## Results

### Results of the literature search

**Figure 1:**
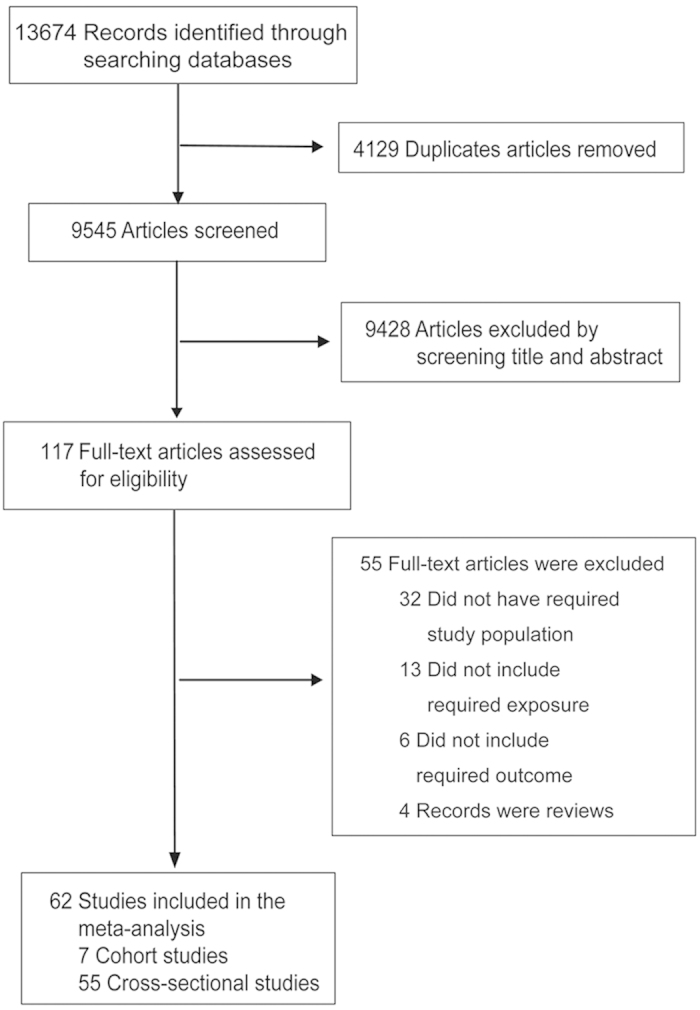
Flowchart for the selection of eligible studies.

The article selection procedure is shown in Fig. 1. Briefly, after excluding 4129 articles due to overlap between search categories, 9545 articles were screened. 9428 articles were excluded by screening title and abstract, and 117 full-text articles were assessed for eligibility. A total of 62 articles fulfilled the inclusion criteria and were included in the meta-analysis, including 7 cohort studies^16^,^17^,^18^,^19^,^20^,^21^,^22^ and 55 cross-sectional studies^10^,^11^,^12^,^13^,^14^,^15^,^23^,^24^,^25^,^26^,^27^,^28^,^29^,^30^,^31^,^32^,^33^,^34^,^35^,^36^,^37^,^38^,^39^,^40^,^41^,^42^,^43^,^44^,^45^,^46^,^47^,^48^,^49^,^50^,^51^,^52^,^53^,^54^,^55^,^56^,^57^,^58^,^59^,^60^,^61^,^62^,^63^,^64^,^65^,^66^,^67^,^68^,^69^,^70^,^71^, which involved approximately 1,305,217 participants (Table S1). A total of 54 estimates from 26 individual studies were available for assessing the association between SEP and risk of childhood overweight, 55 estimates from 28 individual studies were available for assessing the association between SEP and risk of childhood obesity, and 53 estimates from 29 individual studies were available for assessing the association between SEP and risk of childhood overweight including obesity. The higher number of risk estimates compared with number of articles was due to the fact that some studies conducted more than one individual cohort (in different regions, age groups or sexes) and some studies included more than one measure of SEP. The sum of the numbers of the individual studies in three pooled analyses were more than 62 because some studies reported both overweight and obesity outcomes. The quality assessment of the included studies was presented in detail in the supplementary material (Table S2–S3).

### Effect of SEP on overweight and obesity in children

**Figure 2:**
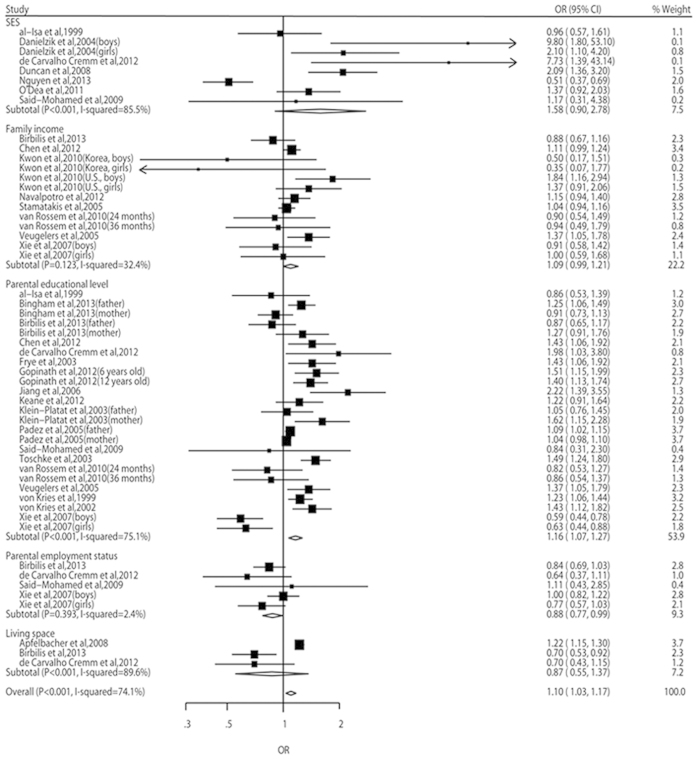
Relative risks and 95% CIs of overweight for the lowest vs highest SEP category in children aged 0–15 years.

**Figure 3:**
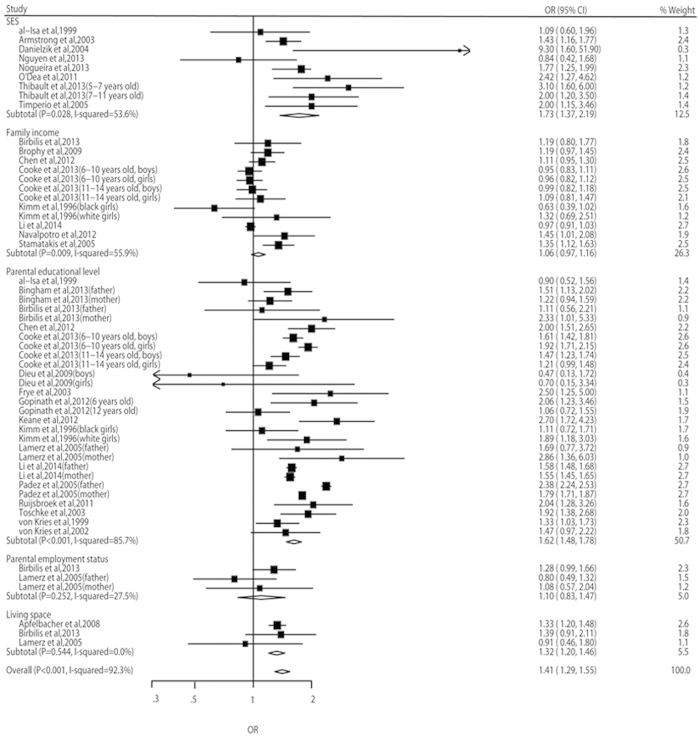
Relative risks and 95% CIs of obesity for the lowest vs highest SEP category in children aged 0–15 years.

**Figure 4:**
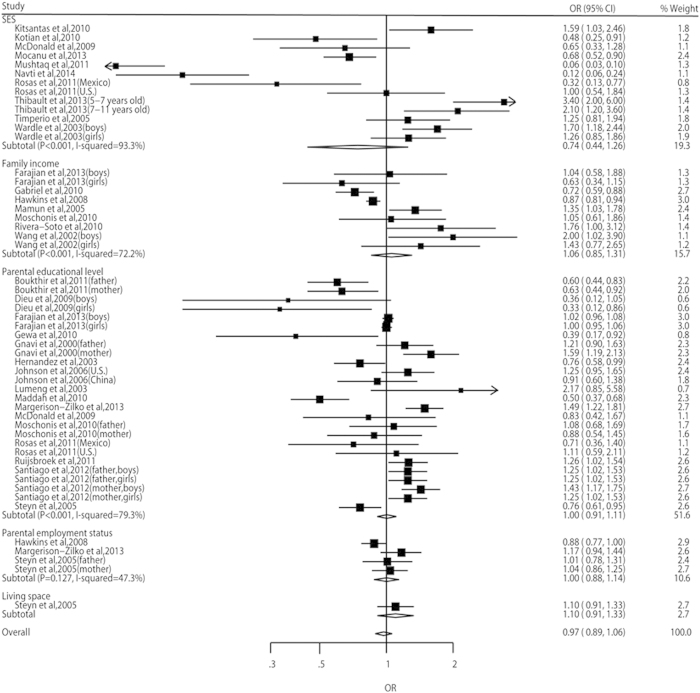
Relative risks and 95% CIs of overweight including obesity for the lowest vs highest SEP category in children aged 0–15 years

In the meta-analyses, the odds of both overweight risk and obesity risk were higher in the children with lowest SEP than in those with highest SEP (OR, 1.10, 95% CI: 1.03–1.17, and OR, 1.41, 95% CI: 1.29–1.55, respectively) (Figs 2 and 3). However, the risk of overweight including obesity didn’t show statistical significance between different SEP groups (OR, 0.97, 95% CI: 0.89–1.06) (Fig. 4). A significant heterogeneity was observed for all three pooled analyses, i.e. overweight (*P* = 0.001, *I*^2^ = 74.1%), obesity (*P* < 0.001, *I*^2^ = 92.3%) and overweight including obesity (*P* < 0.001, *I*^2^ = 85.2%).

### Subgroup analyses

In the subgroup analyses by income level of countries, children with lowest SEP had a 16% (95% CI: 9%−24%) higher risk of overweight, a 43% (95% CI: 30%−58%) higher risk of obesity and a 23% (95% CI: 13%−33%) higher risk of overweight including obesity in high-income countries. However, in middle-income countries, the risks of overweight and obesity didn’t show significant differences between different SEP groups and children with lowest SEP had a 42% (95% CI: 28%−54%) lower risk of overweight including obesity. Only one study from a low-income country was included (Table 1).When we performed sub-groups analyses for different geographical areas, children with lowest SEP had higher risks of overweight, obesity and overweight including obesity in North America, Europe and Oceania. Especially in Oceania, children with lowest SEP had a 50% higher risk of overweight and a 73% higher risk of obesity (OR, 1.50, 95% CI: 1.29–1.73, and OR, 1.73, 95% CI: 1.15–2.59, respectively). But in Asia/Middle East, Latin America and Africa, the risks of overweight and obesity didn’t show significant differences between different SEP groups and children with lowest SEP had lower risk of overweight including obesity (Table 1). When we performed sub-group analyses for different sexes, only studies included both sexes showed higher risks of overweight and obesity in children with lowest SEP, and this may caused by the fact that there were not enough studies conducted in boys and girls separately (Table 1). In other sub-group analyses such as adjustment, publication year and study design, the increased risks of overweight and obesity persisted in the majority of analyses (Table 1). Moreover, significant heterogeneity was present for the majority of sub-group analyses (Table 1).

### Publication bias

Visual assessment of funnel plots (Figure S1) showed that the studies were distributed fairly symmetrically about the combined effect size, which suggests little publication bias. Egger’s regression test and Begg-Mazumdar test also showed that there was no potential publication bias when pooling the results of overweight (Egger’s regression test (p = 0.997), and Begg-Mazumdar test (p = 0.788)), obesity (Egger’s regression test (p = 0.181), and Begg-Mazumdar test (p = 0.141)) and overweight including obesity (Egger’s regression test (p = 0.619), and Begg-Mazumdar test (p = 0.056)).

## Discussion

SEP has been described as inversely related to obesity in adulthood^72^,^73^,^74^. Our findings further suggest that low SEP is associated with a 10% higher risk of overweight and a 41% higher risk of obesity in children aged 0–15 years. However, according to the sub-group analyses by income level of countries, this relationship was only found in high-income countries. In addition, sub-group analyses by geographical areas showed that children with low SEP had higher risks of overweight and obesity only in North America, Europe and Oceania, and the included studies conducted in these areas were all from economic developed countries with high income level. Thus, we concluded that the increased risks were independent of the income levels of countries. Previous studies indicated that overweight and obesity tended to affect more people from a low socio-economic background in developed countries rather than in developing economies^75^,^76^,^77^. This relationship has been further confirmed in children in our quantitative analyses. Higher risks of overweight and obesity in children with lower SEP in developed countries may be related to less access to healthy food and to safe exercise, less interest in weight control, cultural standards of physical effectiveness, and discrimination against socioeconomic advancement^75^,^78^, and insufficient food supply is rare even in families with low SEP. However, situations were different in developing countries and less economic developed areas, where malnutrition and opulence co-exist, food availability remains a daily challenge in populations with low SEP and overweight is subsequently perceived as a sign of weath^77^. These factors may explain why increased risks of childhood overweight and obesity associated with lower SEP were only found in high-income countries and in more economic developed areas.

The pooled analysis found that the risk of overweight including obesity didn’t show statistical significance between different SEP groups. This may be explained by the fact that this pooled analysis involved more studies from middle-income countries and one study from a low-income country, and it is possible that including data on SEP and overweight including obesity from these countries would have changed the picture. However, the sub-group analyses showed that children with low SEP also had a higher risk of overweight including obesity in high-income countries and in more economic developed areas, which was consistent with the overweight and obesity outcomes. Low SEP was even a protective factor for overweight including obesity in middle-income and low-income countries, and also in less economic developed areas.

In adults, the inverse relationship between SEP and obesity was reported to be more pronounced in women than in men^75^,^79^,^80^. However, in our sub-group analyses by sex, the risks of overweight and obesity didn’t show significant differences between SEP groups for both boys and girls, and only boys with low SEP showed a higher risk of overweight including obesity. The inconsistent results for children and adults may due to the fact that children and adults have different patterns on obesity risk classified by SEP, and may also due to the limited studies included in our analyses. Further studies are needed to identify the relationship of SEP and the risks of overweight and obesity for boys and girls separately.

Parental educational level was the most frequently used method as measure of SEP among the included studies. The subtotal point estimates of overweight and obesity risks patterned by parental educational level were greater than other subtotal and overall point estimates. This means that parental educational level was more consistently inversely associated with childhood overweight and obesity than other indicators. This conclusion was also supported by a previous review^8^. As an important socio-economic indicator, parental educational level influences the family’s knowledge and beliefs, and these are considered important for healthy lifestyles and the development of overweight and obesity. However, SEP is a multifactor construct, and parental educational level does not entirely capture the material and financial aspects of socio-economic status. The literature reported on a range of different indicators (e.g. income, educational levels and occupation) as being suitable measures of SEP^81^. Therefore, we included SES, family income, parental educational level, parental employment status and living space as a comprehensive measure of SEP in our meta-analyses.

Some mechanisms may explain the higher risks of overweight and obesity in children with low SEP. Children from lower socio-economic strata may have diets rich in low cost energy dense food^82^,^83^, participate less in physical activity or sports^84^, and have lower awareness of weight control^85^. On the basis of our findings, we advocate that development programmes should be an essential component of overweight and obesity control in children. Public health interventions aimed at relieving economic hardship may reduce the risks of childhood overweight and obesity, especially in the developed countries. Future research should provide more information on the mechanisms of how lower SEP may influence childhood obesity and on the effectiveness of specific public health interventions to combat obesity in children.

The strengths of this meta-analysis are that we reviewed and included a wide range of studies to make a quantitative assessment on SEP and risks of childhood overweight and obesity, as the literature diverges on whether low SEP is a risk factor of overweight and obesity in children, and the pooled results were more precise with more narrow confidence intervals due to the larger sample size, compared with each individual study.

A major limitation of our study is that there were potential heterogeneities in majority of the analyses, and although we did random-effects and sub-group analyses, these are unlikely to have fully accounted for heterogeneity. Another important limitation is that the definitions of overweight and obesity and the classification of SEP may be different across studies, and this may be another explanation of heterogeneity existed among the studies. Overweight and obesity may be defined differently according to anthropometric measurements in different countries as different populations may have different optimal cutoff points in determining overweight and obesity. The measures of SEP such as parental educational level and family income may vary significantly between countries due to differences in country educational and economies systems. However, the SEP groups were divided into two extreme categories, the lowest and the highest, therefore, we assume that we have captured the sense of SEP, irrespective of time and site^86^. In addition, some included studies didn’t make adjustment for other factors or only make adjustment for a few important factors, thus, the risk of overweight or obesity in these studies may be contributed to other factors. In this study, we used SES, family income, parental educational level, parental employment status and living space as measures of SEP. Other indicators such as ownership of assets and neighbourhood environments were also measures of SEP. However, this study was unable to select more SEP indicators for further analysis due to the limitation of the datasets.

In conclusion, this first systematic review and meta-analysis suggests that children with lower SEP had higher risks of overweight and obesity, and the increased risks were independent of the income levels of countries.

## Methods

### Search strategy and eligibility criteria

We followed the guidelines published by the Meta-analysis of Observational Studies in Epidemiology (MOOSE) group to complete the meta-analysis (Table S4)^87^. To identify eligible articles published in English-speaking peer-reviewed journals, we searched Medline, Web of Science, Embase, and the Cochrane Database of Systematic Reviews (1990 to Sep 4, 2014). We selected synonymous terms and used these to develop the search strategy (Panel S1). In addition, we also manually reviewed the references of all retrieved articles and recent reviews to identify relevant studies. We included studies after 1990, since we sought to examine evidence from the period most applicable to the present status of overweight and obesity control. The search strategy was not limited by study design.

The eligible studies should meet the following inclusion criteria: the study population consisted of children aged 0–15 years; the association between SEP and overweight/obesity was assessed; presented risk estimates with confidence intervals or sufficient information to calculated these; and used socio-economic status (SES), family income, parental educational level, parental employment status or living space as individual measures of SEP. Cross-sectional, cohort, and case-control studies were all included in the analysis. Only studies clearly defined overweight or obesity using a recognized standard were included, and studies only reported the body mass index (BMI) classification were excluded from analysis.

### Data extraction

Two investigators (S.W. and Y.D.) independently extracted the following data from each publication: author, country, study design, sample size, age, SEP measure, outcome (overweight or obesity or overweight including obesity), number with overweight or obesity in study, risk estimates with 95% CIs, and factors adjusted for. Countries were classified according to geographical area (North America, Europe, Oceania, Asia/Middle East, Latin America or Africa) and income category according to the World Bank definition (high-income, upper-middle-income, lower-middle-income or low-income country). We extracted estimates for boys and girls separately when possible. If a study reported risk estimates with more than one measure of SEP, each estimate was extracted separately. We included the most adjusted estimate when a study reported more than one risk estimate. The quality of each study was assessed by two investigators (F.W. and J.H.), using methods recommended by Wells and colleagues^88^.

### Statistical analysis

We used odds ratios (ORs) as summary estimates throughout the procedure. We extracted the lowest vs the highest SEP category from each study, using highest SEP as the reference group. The measures of different SEP groups included lowest vs highest SES, lowest vs highest family income, lowest vs highest parental educational level, none vs any of parental employment, and lowest vs highest living space. If presented in a reverse order, we back-calculated the point estimate and 95% CIs. The log ORs from the individual studies and corresponding standard error (SE; calculated from the confidence limits) were used to perform the analysis^86^.

A fixed effects model was used to estimate the pooled ORs and 95% CIs if there was no evidence of heterogeneity; otherwise, a random effect model was used. The *Q*-statistic and *I*-squared (*I*^2^) statistic were used to explore the heterogeneity among studies. For *Q*-statistic, we considered *P* < 0.1 as representative of statistically significant heterogeneity. For *I*^2^ statistic, large *I*^2^ (>50%) suggests substantial heterogeneity among studies. Subgroup analyses were conducted by stratifying the original data sets by the above mentioned study-level factors. Publication bias was visually assessed by funnel plots. Egger’s regression test^89^ and Begg-Mazumdar test^90^ were performed to further assess publication bias. Statistical analyses were conducted using Stata Version 12.0 software (Stata Corp, College Station, TX).

## Additional Information

**How to cite this article**: Wu, S. *et al*. Socio-economic position as an intervention against overweight and obesity in children: a systematic review and meta-analysis. *Sci. Rep*. **5**, 11354; doi: 10.1038/srep11354 (2015).

## Supplementary Material

Supplementary Information

## Figures and Tables

**Table 1:** Sub-group analyses for studies included in the analysis (lowest vs highest SEP category)

Sub-group analysis	Pooled OR (95% CI), *P*-value for the heterogeneity *Q* test, *I*^2^ statistics (%), number of estimates in included studies (*n*)					
	n	Risk estimates of overweight	n	Risk estimates of obesity	n	Risk estimates of overweight including obesity
**Sex**						
Boys	6	1.03 (0.67–1.57); *P* < 0.001, *I*^2^ = 81.6	6	1.24 (0.92–1.65); *P* < 0.001, *I*^2^ = 89.4	7	1.25 (1.01–1.55); *P* < 0.001, *I*^2^ = 77.1
Girls	6	0.96 (0.67–1.39); *P* = 0.005, *I*^2^ = 70.2	7	1.13 (0.81–1.57); *P* < 0.001, *I*^2^ = 91.2	7	1.09 (0.91–1.30); *P* = 0.007, *I*^2^ = 66.4
Combined	42	1.13 (1.06–1.21); *P* < 0.001, *I*^2^ = 72.3	42	1.49 (1.34–1.66); *P* < 0.001, *I*^2^ = 92.5	39	0.90 (0.79–1.03); *P* < 0.001, *I*^2^ = 86.9
**Income**						
High-income	37	1.16 (1.09–1.24); *P* < 0.001, *I*^2^ = 68.9	50	1.43 (1.30–1.58); *P* < 0.001, *I*^2^ = 92.7	32	1.23 (1.13–1.33); *P* < 0.001, *I*^2^ = 76.4
Middle-income	17	0.95 (0.78–1.15); *P* < 0.001, *I*^2^ = 78.6	5	1.14 (0.74–1.77); *P* = 0.002, *I*^2^ = 76.4	20	0.58 (0.46–0.72); *P* < 0.001, *I*^2^ = 87.2
Low	0	−	0	–	1	0.39 (0.17–0.92)
**Geographical area**						
North America	4	1.42 (1.21–1.67); *P* = 0.714, *I*^2^ = 0.0	12	1.22 (1.01–1.47); *P* < 0.001, *I*^2^ = 90.2	8	1.34 (1.19–1.50); *P* = 0.471, *I*^2^ = 0.0
Europe	25	1.11 (1.04–1.19); *P* < 0.001, *I*^2^ = 70.9	32	1.53 (1.36–1.73); *P* < 0.001, *I*^2^ = 93.9	21	1.14 (1.04–1.25); *P* < 0.001, *I*^2^ = 80.9
Oceania	4	1.50 (1.29–1.73); *P* = 0.407, *I*^2^ = 0.0	4	1.73 (1.15–2.59); *P* = 0.056, *I*^2^ = 60.3	4	1.39 (1.13–1.70); *P* = 0.703, *I*^2^ = 0.0
Asia/Middle East	14	0.89 (0.73–1.08); *P* < 0.001, *I*^2^ = 79.6	7	1.12 (0.81–1.54); *P* = 0.006, *I*^2^ = 67.2	6	0.35 (0.16–0.74); *P* < 0.001, *I*^2^ = 90.9
Latin America	4	1.23 (0.58–2.60); *P* = 0.003, *I*^2^ = 78.6	0	–	6	0.72 (0.62–0.83); *P* = 0.609, *I*^2^ = 0.0
Africa	3	1.01 (0.55–1.86); *P* = 0.898, *I*^2^ = 0.0	0	–	8	0.68 (0.51–0.91); *P* < 0.001, *I*^2^ = 87.3
**Adjustment**						
Minimally adjusted	28	1.10 (1.01–1.18); *P* < 0.001, *I*^2^ = 76.9	34	1.42 (1.27–1.60); *P* < 0.001, *I*^2^ = 91.8	31	0.87 (0.73–1.04); *P* < 0.001, *I*^2^ = 87.3
Maximally adjusted	26	1.11 (0.97–1.27); *P* < 0.001, *I*^2^ = 71.5	21	1.39 (1.19–1.62); *P* < 0.001, *I*^2^ = 89.8	22	1.02 (0.93–1.13); *P* < 0.001, *I*^2^ = 81.6
**Publication year**						
< 2010s	27	1.15 (1.06–1.25); *P* < 0.001, *I*^2^ = 76.1	25	1.41 (1.22–1.62); *P* < 0.001, *I*^2^ = 88.6	22	1.06 (0.94–1.19); *P* < 0.001, *I*^2^ = 71.2
≥2010s	27	1.04 (0.93–1.17); *P* < 0.001, *I*^2^ = 72.2	30	1.41 (1.27–1.58); *P* < 0.001, *I*^2^ = 90.4	31	0.90 (0.79–1.03); *P* < 0.001, *I*^2^ = 89.1
**Design**						
Cohort	4	0.87 (0.68–1.11); *P* = 0.986, *I*^2^ = 0.0	4	1.43 (1.07–1.91); *P* < 0.001, *I*^2^ = 98.0	8	1.19 (0.98–1.44); *P* < 0.001, *I*^2^ = 86.4
Cross-sectional	50	1.11 (1.04–1.19); *P* < 0.001, *I*^2^ = 75.6	51	1.41 (1.28–1.56); *P* < 0.001, *I*^2^ = 89.1	45	0.92 (0.83–1.02); *P* < 0.001, *I*^2^ = 85.4
