# Supplementary material for: Socio-economic position as an intervention against overweight and obesity in children: a systematic review and meta-analysis
Source: Sci Rep. 2015 Jun 26;5:11354. doi: 10.1038/srep11354 (PMC4481703; doi:10.1038/srep11354)
Supplement: Supplementary Information [file srep11354-s1.pdf]

Socio-economic position as an intervention against overweight and obesity in children: a systematic review and meta-analysis

Author list:

Shunquan Wu[1, *], Yingying Ding[2, *], Fuquan Wu[3, *], Ruisheng Li[1], Jun Hou[1, †], Panyong Mao[1, †]

***Table S1*: Studies included in the systematic review and meta-analysis**

Study	Study site	Country income level	Study design	n	Participants	SEP measure	Outcome	Number with overweight or obesity in study	OR (95% CI)	Factors adjusted for
al-Isa et al,(28) 1999	Kuwait	High	Cross-sectional	3473	Children aged 3-5 years	SES	Overweight	137	0.96 (0.57-1.61)	Gender, age, governorate, maternal education, birth order, dental status, eating regular meals, and servants
							Obesity	339	1.09 (0.60-1.96)	
						Maternal education	Overweight	137	0.86 (0.53-1.39)	
							Obesity	339	0.90 (0.52-1.56)	
Apfelbacher et al,(29) 2008	Germany	High	Cross-sectional	35434	Children aged 5-7 years	Living space	Overweight	5486	1.22 (1.15-1.30)	None
							Obesity	1534	1.33 (1.20-1.48)	
Armstrong et al,(30) 2003	United Kingdom	High	Cross-sectional	74500	Children aged 39-42 months	SES	Obesity	2788	1.43 (1.16-1.77)	Birth weight
Bingham et al,(31) 2013	Portugal	High	Cross-sectional	17136	Children aged 3-10 years	Maternal education	Overweight	3382	0.91 (0.73-1.13)	Sex and age
							Obesity	1404	1.22 (0.94-1.59)	
						Paternal education	Overweight	3382	1.25 (1.06-1.49)	
							Obesity	1404	1.51 (1.13-2.02)	
Birbilis et al,(32) 2013	Greece	High	Cross-sectional	2294	Children aged 9-13 years	Paternal education	Overweight	700	0.87 (0.65-1.17)	None
							Obesity	266	1.11 (0.56-2.21)	Dietary energy intake and physical activity levels
						Maternal education	Overweight	700	1.27 (0.91-1.76)	None

							Obesity	266	2.33 (1.01-5.33)	Dietary energy intake and physical activity levels
						Family income	Overweight	700	0.88 (0.67-1.16)	None
							Obesity	266	1.19 (0.80-1.77)	
						Living space	Overweight	700	0.70 (0.53-0.92)	
							Obesity	266	1.39 (0.91-2.11)	
						Maternal employment status	Overweight	700	0.84 (0.69-1.03)	
							Obesity	266	1.28 (0.99-1.66)	
Boukthir et al,(33) 2011	Tunisia	Upper middle	Cross-sectional	1335	Children aged 6-12 years	Maternal education	Overweight including obesity	264	0.63 (0.44-0.92)	None
						Paternal education	Overweight including obesity	264	0.60 (0.44-0.83)	
Brophy et al,(34) 2009	United Kingdom	High	Cross-sectional	17561	Children aged 5 years	Family income	Obesity	789	1.19 (0.97-1.45)	Sedentary behaviour, birth weight, and family behaviours.
Chen et al,(35) 2012	Taiwan	Upper middle	Cross-sectional	7930	Children aged 9-14 years	Parental education	Overweight	2443	1.43 (1.06-1.92)	None
							Obesity	1321	2.00 (1.51-2.65)	
						Family income	Overweight	2443	1.11 (0.99-1.24)	
							Obesity	1321	1.11 (0.95-1.30)	
Cooke et al,(36) 2013	Canada	High	Cross-sectional	1186	Children aged 6-10 years	Parental education	Obesity	319	1.61 (1.42-1.81)*	Not reported
									1.92 (1.71-2.15)**	

						Family income	Obesity	319	0.95 (0.83-1.01)*	
									0.96 (0.82-1.12)**	
				2874	Children aged 11-14 years	Parental education	Obesity	339	1.47 (1.23-1.74)*	
									1.21 (0.99-1.48)**	
						Family income	Obesity		0.99 (0.82-1.18)*	
									1.09 (0.81-1.47)**	
Danielzik et al,(10) 2004	Germany	High	Cross-sectional	2631	Children aged 5-7 years	SES	Overweight	161	9.80 (1.80-53.10)*	All family-, environment- and development-related determinants
									2.10 (1.10-4.20)**	
							Obesity	106	9.30 (1.60-51.90)*	
de Carvalho Cremm et al,(37) 2012	Brazil	Upper middle	Cross-sectional	302	Children under 6 years of age	Living space	Overweight	107	0.70 (0.43-1.15)	None
						SES	Overweight	107	7.73 (1.39-43.14)	Food intake, level of physical activity, economic status, individual characteristics, and maternal age
				229	Children aged 6-10 years	Maternal education	Overweight	89	1.98 (1.03-3.80)	
						Maternal employment status	Overweight	98	0.64 (0.37-1.11)	None
Dieu et al,(38) 2009	Vietnam	Lower middle	Cross-sectional	1162	Children aged 4-5 years	Parental education	Obesity	Not reported	0.47 (0.13-1.72)*	Age, district, and parental overweight
									0.70 (0.15-3.34)**	
						Parental education	Overweight including	352	0.36 (0.12-1.05)*	

							obesity			
									0.33 (0.12-0.86)**	
Duncan et al,(39) 2008	New Zealand	High	Cross-sectional	1033	Children aged 5-11 years	SES	Overweight	196	2.09 (1.36-3.20)	Sex, age, ethnicity, physical activity, active transport, sports participation, breakfast, bought lunch, fast food, sugary drink, weekday sleep, and weekend sleep
Farajian et al,(40) 2013	Greece	High	Cross-sectional	2315	Children aged 10-12 years	Paternal education	Overweight including obesity	934	1.02 (0.96-1.08)*	Age, gender, maternal age, paternal type of occupation, place of residence, annual
									1.00 (0.95-1.06)**	family income and parental BMI classification
						Family income	Overweight including obesity	934	1.04 (0.58-1.88)*	Age, gender, maternal age, Paternal type of occupation, paternal educational level,
									0.63 (0.34-1.15)**	place of residence, and parental BMI classification
Frye et al,(41) 2003	Germany	High	Cross-sectional	7632	Children aged 5-14 years	Parental education	Overweight	432	1.43 (1.06-1.92)	Age, sex and survey
							Obesity	124	2.50 (1.25-5.00)	
Gabriel et al,(42) 2010	Brazil	Upper middle	Cross-sectional	4964	Children aged 6-10 years	Family income	Overweight including obesity	1067	0.72 (0.59-0.88)	Maternal level of education, paternal level of education, maternal age, Paternal age, paternal BMI, birth weight,

										birth length, and age of foods introduction
Gewa,(43) 2010	Kenya	Low	Cross-sectional	1443	Children aged 3-5 years	Maternal education	Overweight including obesity	257	0.39 (0.17-0.92)	None
Gnavi et al,(44) 2000	Italy	High	Cross-sectional	1420	Children aged 10-11 years	Maternal education	Overweight including obesity	327	1.59 (1.19-2.13)	Parental area of birth, maternal age and school district
						Paternal education	Overweight including obesity	327	1.21 (0.90-1.63)	
Gopinath et al,(45) 2012	Australia	High	Cross-sectional	1741	Children aged 6 years	Parental education	Overweight	225	1.51 (1.15-1.99)	Age, sex and ethnicity
							Obesity	103	2.06 (1.23-3.46)	
				2353	Children aged 12 years	Parental education	Overweight	480	1.40 (1.13-1.74)	
							Obesity	193	1.06 (0.72-1.55)	
Hawkins et al,(21) 2008	United Kingdom	High	Cohort	13113	Children aged 3 years	Maternal employment status	Overweight including obesity	3085	0.88 (0.77-1.00)	Maternal ethnic group, highest academic qualification, age at first live birth, lone motherhood status, maternal pre-pregnancy body size, smoked during pregnancy, birth weight, breastfeeding duration, introduction of

										solid foods, television viewing daily and who primarily cooks the main meal
						Family income	Overweight including obesity	3085	0.87 (0.81-0.94)	Maternal hours worked and duration, partner hours worked and duration, maternal ethnic group, highest academic qualification, age at first live birth, lone motherhood status, maternal pre-pregnancy body size, smoked during pregnancy, birth weight, breastfeeding duration, introduction of solid foods, television viewing daily and who primarily cooks the main meal
Hernandez et al,(46) 2003	Mexico	Upper middle	Cross-sectional	10901	Children aged 5-11 years	Maternal education	Overweight including obesity	2126	0.76 (0.58-0.99)	Region, zone of residence, sex, indigenous ethnicity, socio-economic status and age
Jiang et al,(47) 2006	China	Upper middle	Cross-sectional	930	Children aged 2-6 years	Maternal education	Overweight	100	2.22 (1.39-3.55)	Children's age, gender, family income and

										kindergarten
Johnson et al,(48) 2006	United States (California)	High	Cross-sectional	1772	Children aged 12-13 years	Parental education	Overweight including obesity	788	1.25 (0.95-1.65)	Gender, age, residence and ethnicity
	China (Wuhan)	Upper middle	Cross-sectional	1896	Children aged 12-13 years	Parental education	Overweight including obesity	228	0.91 (0.60-1.38)	Gender, age and residence
Keane et al,(49) 2012	Ireland	High	Cross-sectional	8136	Children aged 9 years	Maternal education	Overweight	1545	1.22 (0.91-1.64)	Study child's gender, study child has siblings, household class, household income and parent weight status
						Maternal education	Obesity	471	2.70 (1.72-4.23)	
Kimm et al,(50) 1996	United States	High	Cross-sectional	1213	Black girls aged 9-10 years	Family income	Obesity	371	0.63 (0.39-1.02)	Maximum education, single or two parents, TV viewing and total caloric intake
						Parental education	Obesity	371	1.11 (0.72-1.71)	Income level, single or two parents, TV viewing and total caloric intake
				1166	White girls aged 9-10 years	Family income	Obesity	225	1.32 (0.69-2.51)	Maximum education, single or two parents, TV viewing and total caloric intake
						Parental education	Obesity	225	1.89 (1.18-3.03)	Income level, single or two parents, TV viewing and total caloric intake
Kitsantas et	United	High	Cross-	6540	Children aged	SES	Overweight	Not reported	1.59 (1.03-2.46)	Not reported

al,(12) 2010	States		sectional		4 years		including obesity			
Klein-Platat et al,(51) 2003	France	High	Cross-sectional	3436	Children aged 12 years	Maternal education	Overweight	Not reported	1.62 (1.15-2.28)	Maternal and Paternal obesity
						Paternal education	Overweight	Not reported	1.05 (0.76-1.45)	
Kotian et al,(15) 2010	India	Lower middle	Cross-sectional	900	Children aged 12-15 years	SES	Overweight including obesity	132	0.48 (0.25-0.91)	Not reported
Kwon et al,(52) 2010	Korea	High	Cross-sectional	2117	Children aged 7-12 years	Family income	Overweight	313	0.50 (0.17-1.51)*<br>0.35 (0.07-1.77)**	TV viewing, frequency of dining out, computer usage, energy from carbohydrate, energy from protein, energy, energy from fat, parental obesity, and parental education level.
	United States	High	Cross-sectional	3016	Children aged 7-12 years	Family income	Overweight	393	1.84 (1.16-2.94)*<br>1.37 (0.91-2.06)**	TV viewing, frequency of dining out, computer usage, energy from carbohydrate, and energy from protein
Lamerz et al,(53) 2005	Germany	High	Cross-sectional	1827	Children aged 6 years	Maternal education	Obesity	Not reported	2.86 (1.36-6.03)	Gender, maternal BMI, paternal BMI, paternal education, maternal employment, paternal employment, living space and single parent
						Paternal education	Obesity	Not reported	1.69 (0.77-3.72)	
						Maternal employment	Obesity	Not reported	1.08 (0.57-2.04)	
						Paternal employment	Obesity	Not reported	0.80 (0.49-1.32)	
						Living space	Obesity	Not reported	0.91 (0.46-1.80)	
Li et al,(22)	Sweden	High	Cohort	94806	Children aged	Family income	Obesity	10779	0.97 (0.91-1.03)	Neighbourhood-level

2014				2	0-14 years					deprivation, sex, age, and
						Maternal education	Obesity	10779	1.55 (1.45-1.65)	family- and individual-level
						Paternal education	Obesity	10779	1.58 (1.48-1.68)	socio-demographic variables.
Lumeng et al,(23) 2003	United States	High	Cohort	755	Children aged 8-11 years	Maternal education	Overweight including obesity	90	2.17 (0.85-5.58)	Sex, ethnicity/race, SES, maternal marital status, maternal depressive symptom, maternal BMI, cognitive stimulation score, maternal smoking status, use of behavior-modifying medication, hours of TV per day, and history of academic grade retention
Maddah et al,(54) 2010	Iran	Upper middle	Cross-sectional	6635	Children aged 6-11 years	Maternal education	Overweight including obesity	1230	0.50 (0.37-0.68)	Age, sex, television viewing, birth rank, maternal employment, parental overweight/obesity, walking, skipping breakfast, and birth weight
Mamun et al,(55) 2005	Australia	High	Cross-sectional	3681	Children aged 14 years	Family income	Overweight including obesity	969	1.35 (1.03-1.78)	Age
Margerison-Zilko et al,(24) 2013	United States	High	Cohort	5613	Children aged 4-14 years	Maternal education	Overweight including obesity	2301	1.49 (1.22-1.81)	Gender, race/ethnicity, age at baseline, maternal baseline age, maternal

						Maternal employment	Overweight including obesity	2301	1.17 (0.94-1.44)	education, maternal BMI, maternal marital status, and maternal employment status
McDonald et al,(56) 2009	Colombia	Upper middle	Cross-sectional	3075	Children aged 5-12 years	Maternal education	Overweight including obesity	341	0.83 (0.42-1.67)	Sex, age, stunting, maternal BMI, parity, and number of household assets
						SES	Overweight including obesity	341	0.65 (0.33-1.28)	
Mocanu,(57) 2013	Romania	Upper middle	Cross-sectional	3444	Children aged 6-10 years	SES	Overweight including obesity	816	0.68 (0.52-0.90)	Sex and age
Moschonis et al,(58) 2010	Greece	High	Cross-sectional	729	Children aged 9-13 years	Family income	Overweight including obesity	297	1.05 (0.61-1.86)	Annual family income, parent's nationality, residence ownership, child's primary caregiver, popularity score, gender and for the clustering effect of schools
						Maternal education	Overweight including obesity	297	0.88 (0.54-1.45)	
						Paternal education	Overweight including obesity	297	1.08 (0.68-1.69)	
Mushtaq et al,(59) 2011	Pakistan	Lower middle	Cross-sectional	1860	Children aged 5-12 years	SES	Overweight including obesity	316	0.06 (0.03-0.10)	Age and gender
Navalpotro et al,(60) 2012	Spain	High	Cross-sectional	4529	Children aged 6-15 years	Family income	Overweight	1502	1.15 (0.94-1.40)	Age, sex, socioeconomic position, diet, physical

							Obesity	330	1.45 (1.01-2.08)	inactivity and watching TV
Navti et al,(13) 2014	Cameroon	Lower middle	Cross-sectional	557	Children aged 5-12 years	SES	Overweight including obesity	97	0.12 (0.06-0.24)	Age and gender
Nguyen et al,(14) 2013	Vietnam	Lower middle	Cross-sectional	1989	Children aged 11-14 years	SES	Overweight	354	0.51 (0.37-0.69)	None
							Obesity	64	0.84 (0.42-1.68)	
Nogueira et al,(61) 2013	Portugal	High	Cross-sectional	1885	Children aged 3-10 years	SES	Obesity	Not reported	1.77 (1.25-1.99)	Gender, age and clustering of children in schools
O'Dea et al,(62) 2011	Australia	High	Cross-sectional	1239	Children aged 9-12 years	SES	Overweight	190	1.37 (0.92-2.03)	Age and gender
							Obesity	65	2.42 (1.27-4.62)	
Padez et al,(63) 2005	Portugal	High	Cross-sectional	4511	Children aged 7-9.5 years	Maternal education	Overweight	914	1.04 (0.98-1.10)	Age and sex
							Obesity	510	1.79 (1.71-1.87)	
						Paternal education	Overweight	914	1.09 (1.02-1.15)	
							Obesity	510	2.38 (2.24-2.53)	
Rivera-Soto et al,(64) 2010	United States	High	Cross-sectional	250	Children aged 6-11 years	Family income	Overweight including obesity	95	1.76 (1.00-3.12)	None
Rosas et al,(65) 2011	Mexico	Upper middle	Cross-sectional	316	Children aged 5 years	Maternal education	Overweight including obesity	47	0.71 (0.36-1.40)	None
						SES	Overweight including obesity	47	0.32 (0.13-0.77)	Maternal weight status, sex, and household food insecurity

	United States	High	Cross-sectional	287	Children aged 5 years	Maternal education	Overweight including obesity	153	1.11 (0.59-2.11)	None
						SES	Overweight including obesity	153	1.00 (0.54-1.84)	Maternal weight status, sex, and household food insecurity
Ruijsbroek et al,(25) 2011	Netherlands	High	Cohort	3963	Children aged 0-8 years	Maternal education	Obesity	91	2.04 (1.28-3.26)	Children's sex, mother smoking during pregnancy, mother smoking in the home, breastfeeding and day-care centre attendance
							Overweight including obesity	567	1.26 (1.02-1.54)	Children's sex, mother smoking during pregnancy, mother smoking in the home and day care centre attendance
Said-Mohamed et al,(66) 2009	Cameroon	Lower middle	Cross-sectional	169	Children aged 24-59 months	SES	Overweight	45	1.17 (0.31-4.38)	Not reported
						Maternal education	Overweight	45	0.84 (0.31-2.30)	
						Maternal employment	Overweight	45	1.11 (0.43-2.85)	
Santiago et al,(67) 2012	Spain	High	Cross-sectional	3101	Children aged 6-12 years	Maternal education	Overweight including obesity	909	1.43 (1.17-1.75)*<br>1.25 (1.02-1.53)**	None
						Paternal education	Overweight including	909	1.25 (1.02-1.53)*	Age, birth weight, number of siblings, father obese and

							obesity			mother obese
									1.25 (1.02-1.53)**	None
Stamatakis et al,(68) 2005	United Kingdom	High	Cross-sectional	11185	Children aged 5-10 years	Family income	Overweight	2331	1.04 (0.94-1.16)	Time point, social class and sex
							Obesity	590	1.35 (1.12-1.63)	
Steyn et al,(69) 2005	South Africa	Upper middle	Cross-sectional	2200	Children aged 1-9 years	Living space	Overweight including	229	1.10 (0.91-1.33)	Age, gender and urban/rural residence
						Maternal education	obesity	229	0.76 (0.61-0.95)	
						Maternal employment		229	1.04 (0.86-1.25)	
						Paternal employment		229	1.01 (0.78-1.31)	
Thibault et al,(11) 2013	France	High	Cross-sectional	4048	Children aged 5-7 years	SES	Overweight including obesity	383	3.40 (2.00-6.00)	Gender, intake of breakfast or afternoon meal, sedentary activity, low SES
							Obesity	90	3.10 (1.60-6.00)	status and eating at the canteen
			Cross-sectional	3619	Children aged 7-11 years	SES	Overweight including obesity	565	2.10 (1.20-3.60)	Gender, morning snack, sedentary activity, low SES status and geographical area
							Obesity	105	2.00 (1.20-3.50)	
Timperio et al,(70) 2005	Australia	High	Cross-sectional	916	Children aged 10-12 years	SES	Obesity	61	2.00 (1.15-3.46)	Sex, cluster of children by school, and family owning
							Overweight including obesity	264	1.25 (0.81-1.94)	of car
Toschke et al,(71) 2003	Germany	High	Cross-sectional	4974	Children aged 5-6 years	Parental education	Overweight	Not reported	1.49 (1.24-1.80)	None

							Obesity	Not reported	1.92 (1.38-2.68)	
van Rossem et al,(26) 2010	Netherlands	High	Cohort	2954	Children aged 24 and 36 months	Maternal education	Overweight (24 months)	Not reported	0.82 (0.53-1.27)	Age, sex, smoking during pregnancy, parental BMI, birth weight, gestational
							Overweight (36 months)	Not reported	0.86 (0.54-1.37)	age, breastfeeding, and infant BMI SDS change
						Family income	Overweight (24 months)	Not reported	0.90 (0.54-1.49)	between 1 to 6 months after birth
							Overweight (36 months)	Not reported	0.94 (0.49-1.79)	
Veugelers et al,(72) 2005	Canada	High	Cross-sectional	4298	Children aged 10-11 years	Parental education	Overweight	1414	1.37 (1.05-1.79)	Lunch and family supper in dietary habits, physical activities, neighbourhood income in sociodemographic factors, and frequency of physical education classes in school-based factors
						Family income	Overweight	1414	1.37 (1.05-1.78)	Parental education and neighbourhood income
von Kries et al,(73) 1999	Germany	High	Cross-sectional	9357	Children aged 5-6 years	Parental education	Overweight	Not reported	1.23 (1.06-1.44)	Maternal smoking, birth weight, own bedroom, and
							Obesity	Not reported	1.33 (1.03-1.73)	frequent consumption of butter
von Kries et al,(74) 2002	Germany	High	Cross-sectional	6483	Children aged 5-7 years	Maternal education	Overweight	Not reported	1.43 (1.12-1.82)	Maternal smoking during pregnancy, parental BMI,

							Obesity	Not reported	1.47 (0.97-2.22)	birth weight, weight gain, watching TV or play video games, sports activities, breastfeeding, solid foods introduced before month 4, given a bottle containing milk or tea with carbohydrates to sleep, and eating snacks while watching TV
Wang et al,(75) 2002	Australia	High	Cross-sectional	1354	Children aged 7-15 years	Family income	Overweight including obesity	328	2.00 (1.02-3.90)*<br>1.43 (0.77-2.65)**	Age
Wardle et al,(27) 2003	United Kingdom	High	Cohort	4320	Children aged 11-12 years	SES	Overweight including obesity	1045	1.70 (1.18-2.44)*<br>1.26 (0.85-1.86)**	Age, ethnicity and clustering within schools
Xie et al,(76) 2007	China	Upper middle	Cross-sectional	6863	Children aged 12-14 years	Parental education	Overweight	745	0.59 (0.44-0.78)*<br>0.63 (0.44-0.88)**	City residence, age, puberty, family income, parental education, parental employment, and ownership of electronics
						Family income	Overweight	745	0.91 (0.58-1.42)*<br>1.00 (0.59-1.68)**	
						Parental employment	Overweight	745	1.00 (0.82-1.22)*<br>0.77 (0.57-1.03)**	

OR=odds ratio. CI= confidence interval. SEP= socio-economic position. SES= socio-economic status.

* ORs and 95% CIs for boys.

** ORs and 95% CIs for girls.

Table S2: **Quality assessment of the included studies (cohort studies)**

Study	Selection				Comparability	Outcome			Overall quality assessment score (of a maximum of 9)
	Representativeness of the exposed cohort	Selection of the non exposed cohort	Ascertainment of exposure	Demonstration that outcome of interest was not present at start of study	Comparability of cohorts on the basis of the design or analysis	Assessment of outcome	Was follow-up long enough for outcomes to occur	Adequacy of follow up of cohorts	
Hawkins et al, 2008	* Truly representative of the average child in the community	* Drawn from the same community as the exposed cohort	* Structured interview	The study didn't demonstrate that overweight/obesity was not present at start of study	** The study controls for maternal hours worked and duration, partner hours worked and duration, maternal ethnic group, highest academic qualification, age at first live birth, lone motherhood status, maternal pre-pregnancy body size, smoked during	* Independent blind assessment	* The study select an adequate follow up period for outcome of interest	* Subjects lost to follow up unlikely to introduce bias (80% follow up)	8

					pregnancy, birthweight, breastfeeding duration, introduction of solid foods, television viewing daily and who primarily cooks the main meal				
Li et al, 2014	* Truly representative of the average child in the community	* Drawn from the same communit y as the exposed cohort	* Structured interview	The study didn't demonstrate that obesity was not present at start of study	** The study controls for neighbourhood-l evel deprivation, sex, age, and family- and individual-level socio-demograp hic variabels.	* Independent blind assessment	* The study select an adequate follow up period for outcome of interest	Not reported	7
Lumeng et al, 2003	* Truly representative of the average child in the community	* Drawn from the same communit y as the exposed	* Structured interview	The study didn't demonstrate that overweight/obe sity was not	** The study controls for sex, ethnicity/race, SES, maternal marital status, maternal	* Independent blind assessment	* The study select an adequate follow up period for outcome of	Not reported	7

		cohort		present at start of study	depressive symptom, maternal BMI, cognitive stimulation score, maternal smoking status, use of behavior-modifying medication, hours of TV per day, and history of academic grade retention		interest		
Margerison-Zilko et al, 2013	* Truly representative of the average child in the community	* Drawn from the same community as the exposed cohort	* Structured interview	* The study demonstrate that obesity was not present at start of study	** The study controls for gender, race/ethnicity, age at baseline, maternal baseline age, maternal BMI, maternal marital status, and maternal education/mater	* Independent blind assessment	* The study select an adequate follow up period for outcome of interest	* Subjects lost to follow up unlikely to introduce bias (98% follow up)	9

					nal employment status				
Ruijsbroek et al, 2011	* Truly representative of the average child in the community	* Drawn from the same communit y as the exposed cohort	* Structured interview	The study didn't demonstrate that overweight/obe sity was not present at start of study	** The study controls for children's sex, mother smoking during pregnancy, mother smoking in the home, breastfeeding and day-care centre attendance	Self report	* The study select an adequate follow up period for outcome of interest	* Subjects lost to follow up unlikely to introduce bias (92% follow up)	7
van Rossem et al, 2010	No description of the derivation of the cohort	* Drawn from the same communit y as the exposed cohort	* Structured interview	* The study demonstrate that overweight was not present at start of study	** The study controls for age, sex, smoking during pregnancy, parental BMI, birth weight, gestational age, breastfeeding, and infant BMI SDS change between 1 to 8	* Independent blind assessment	The study select an not adequate follow up period for outcome of interest	* Subjects lost to follow up unlikely to introduce bias (76% follow up)	7

					months after birth				
Wardle et al, 2003	* Truly representative of the average child in the community	* Drawn from the same communit y as the exposed cohort	* Structured interview	The study didn't demonstrate that overweight/obe sity was not present at start of study	* The study controls for age, ethnicity and clustering within schools	* Independent blind assessment	* The study select an adequate follow up period for outcome of interest	* Subjects lost to follow up unlikely to introduce bias (84% follow up)	7

Table S3: **Quality assessment of the included studies (cross-sectional studies)**

Study	Selection		Comparability	Exposure	Overall quality assessment score (of a maximum of 5)
	Representativeness of the sample	Ascertainment of exposure	Comparability of groups on the basis of the design or analysis	Assessment of outcome	
al-Isa et al, 1999	* Truly representative of the average child in the community	* Structured interview	** Study controls for gender, age, governorate, SES/mother's education, birth order, dental status, eating regular meals, and servants	* Independent blind assessment	5
Apfelbacher et al, 2008	* Truly representative of the average child in the community	* Structured interview	Study does not control for other factors	* Independent blind assessment	3
Armstrong et al, 2003	* Truly representative of the average child in the community	* Structured interview	* Study controls for birth weight	* Independent blind assessment	4
Bingham et al, 2013	* Truly representative of the average child in the community	* Structured interview	* Study controls for sex and age	* Independent blind assessment	4
Birbilis et al, 2013	* Truly representative of the average child in the community	* Structured interview	Study does not control for other factors in most analyses	* Independent blind assessment	3
Boukthir et al, 2011	* Somewhat representative of the average child in the community	* Structured interview	Study does not control for other factors in most analyses	* Independent blind assessment	3
Brophy et al, 2009	* Truly representative of the average child in the community	* Structured interview	* Study controls for sedentary behaviour, birth weight, and family	* Independent blind assessment	4

			behaviours.		
Chen et al, 2012	* Truly representative of the average child in the community	* Structured interview	Study does not control for other factors in most analyses	* Independent blind assessment	3
Cooke et al, 2013	* Truly representative of the average child in the community	* Structured interview	** Study controls for all of the identified covariates	* Independent blind assessment	5
Danielzik et al, 2004	* Truly representative of the average child in the community	* Structured interview	** Study controls for all family-, environment- and development-related determinants	* Independent blind assessment	5
de Carvalho Cremm et al, 2012	* Truly representative of the average child in the community	* Structured interview	* Study controls for food intake, level of physical activity, economic status, individual characteristics, and mother's age in most analyses	* Independent blind assessment	4
Dieu et al, 2009	* Truly representative of the average child in the community	* Structured interview	* Study controls for age, district, and parental overweight	* Independent blind assessment	4
Duncan et al, 2008	* Truly representative of the average child in the community	* Structured interview	** Study controls for sex, age, ethnicity, physical activity, active transport, sports participation, breakfast, bought lunch, fast food, sugary drink,	* Independent blind assessment	5

			weekday sleep, and weekend sleep		
Farajian et al, 2013	* Truly representative of the average child in the community	* Structured interview	** Study controls for age, gender, mother's age, father's type of occupation, place of residence, father’s educational level/annual family income, and parental BMI classification	* Independent blind assessment	5
Frye et al, 2003	* Somewhat representative of the average child in the community	* Structured interview	* Study controls for age, sex and survey	* Independent blind assessment	4
Gabriel et al, 2010	* Truly representative of the average child in the community	* Structured interview	** Study controls for mother's level of education, father's level of educaiton, mother's age, father's age, father's BMI, birth weight, birth length, and age of foods introduction	* Independent blind assessment	5
Gewa, 2010	* Truly representative of the average child in the community	* Structured interview	Study does not control for other factors	* Independent blind assessment	3
Gnavi et al, 2000	* Truly representative of the average child in the community	* Structured interview	* Study controls for parental area of birth, mother's age and school	* Independent blind assessment	4

			district		
Gopinath et al, 2012	* Truly representative of the average child in the community	* Structured interview	* Study controls for age, sex and ethnicity	* Independent blind assessment	4
Hernandez et al, 2003	* Truly representative of the average child in the community	* Structured interview	* Study controls for region, zone of residence, sex, indigenous ethnicity, so io-economic status and age	* Independent blind assessment	4
Jiang et al, 2006	* Somewhat representative of the average child in the community	* Structured interview	* Study controls for children's age, gender, family income and kindergarten	* Independent blind assessment	4
Johnson et al, 2006	* Truly representative of the average child in the community	* Structured interview	* Study controls for gender, age and residence	* Independent blind assessment	4
Keane et al, 2012	* Truly representative of the average child in the community	* Structured interview	** Study controls for study child's gender, study child has siblings, household class, household income and parent weight status	* Independent blind assessment	5
Kimm et al, 1996	* Truly representative of the average child in the community	* Structured interview	** Study controls for maximum education/income level, single or two parents, TV viewing and total caloric intake	* Independent blind assessment	5

Kitsantas et al, 2010	* Truly representative of the average child in the community	* Structured interview	Study does not report the controlled factors	* Independent blind assessment	3
Klein-Platat et al, 2003	* Truly representative of the average child in the community	* Structured interview	* Study controls for mother's and father's obesity	* Independent blind assessment	4
Kotian et al, 2010	* Truly representative of the average child in the community	* Structured interview	Study does not report the controlled factors	* Independent blind assessment	3
Kwon et al, 2010	* Truly representative of the average child in the community	* Structured interview	** Study controls for TV viewing, frequency of dining out, computer usage, energy from carbohydrate, energy from protein, energy, energy from fat, parental obesity, and parental education level.	* Independent blind assessment	5
Lamerz et al, 2005	* Somewhat representative of the average child in the community	* Structured interview	** Study controls for gender, maternal BMI, paternal BMI, maternal education, paternal education, maternal employment, paternal employment, living space and single parent	* Independent blind assessment	5
Maddah et al, 2010	* Somewhat representative of the average child in the community	* Structured interview	** Study controls for age, sex, television	* Independent blind assessment	5

			viewing, birth rank, mother's employment, parental overweight/obesity, walking, skipping breakfast, and birth weight		
Mamun et al, 2005	No description	* Structured interview	* Study controls for age	* Independent blind assessment	3
McDonald et al, 2009	* Truly representative of the average child in the community	* Structured interview	** Study controls for sex, age, stunting, maternal BMI, parity, and number of household assets	* Independent blind assessment	5
Mocanu, 2013	* Truly representative of the average child in the community	* Structured interview	* Study controls for sex and age	* Independent blind assessment	4
Moschonis et al, 2010	* Truly representative of the average child in the community	* Structured interview	** Study controls for annual family income, parent's nationality, residence ownership, child's primary caregiver, popularity score, gender and for the clustering effect of schools	* Independent blind assessment	5
Mushtaq et al, 2011	* Truly representative of the average child in the community	* Structured interview	* Study controls for age and gender	* Independent blind assessment	4
Navalpotro et al,	* Truly representative of the	* Structured interview	** Study controls for age,	Not reported	4

2012	average child in the community		sex, socioeconomic position, diet, physical inactivity and watching TV		
Navti et al, 2014	* Somewhat representative of the average child in the community	* Structured interview	* Study controls for age and gender	* Independent blind assessment	4
Nguyen et al, 2013	* Truly representative of the average child in the community	* Structured interview	Study does not control for other factors in most analyses	* Independent blind assessment	3
Nogueira et al, 2013	No description	* Structured interview	* Study controls for gender, age and clustering of children in schools	* Independent blind assessment	3
O'Dea et al, 2011	* Somewhat representative of the average child in the community	* Structured interview	* Study controls for age and gender	* Independent blind assessment	4
Padez et al, 2005	* Truly representative of the average child in the community	* Structured interview	* Study controls for age and sex	* Independent blind assessment	4
Rivera-Soto et al, 2010	* Truly representative of the average child in the community	Not reported	Study does not control for other factors in most analyses	* Independent blind assessment	2
Rosas et al, 2011	* Somewhat representative of the average child in the community	* Structured interview	Study does not control for other factors in most analyses in most analyses	* Independent blind assessment	3
Said-Mohamed et al, 2009	Potential for selection biases	* Structured interview	Study does not report the controlled facotrs	* Independent blind assessment	2
Santiago et al,	* Somewhat representative of the	* Structured interview	Study does not control	Self report	2

2012	average child in the community		for other factors in most analyses		
Stamatakis et al, 2005	* Truly representative of the average child in the community	* Structured interview	* Study controls for age, gender and urban/rural residence	* Independent blind assessment	4
Steyn et al, 2005	* Truly representative of the average child in the community	* Structured interview	* Study controls for time point, social class and sex	* Independent blind assessment	4
Thibault et al, 2013	* Truly representative of the average child in the community	* Structured interview	** Study controls for gender, intake of breakfast or afternoon meal/morning snack, sedentary activity, low SES status and eating at the canteen/geographical area	* Independent blind assessment	5
Timperio et al, 2005	* Truly representative of the average child in the community	* Structured interview	* Study controls for sex,cluster of children by school, and family owning of car,	* Independent blind assessment	4
Toschke et al, 2003	* Somewhat representative of the average child in the community	* Structured interview	Study does not control for other factors in most analyses	* Independent blind assessment	3
Veugelers et al, 2005	* Somewhat representative of the average child in the community	* Structured interview	** Study controls for lunch and family supper in dietary habits, physical activities, parental	* Independent blind assessment	5

			education, neighbourhood income in sociodemographic factors, and frequency of physical education classes in school-based factors		
von Kries et al, 1999	* Somewhat representative of the average child in the community	* Structured interview	** Study controls for maternal smoking, birth weight, own bedroom, and frequent consumption of butter	* Independent blind assessment	5
von Kries et al, 2002	* Somewhat representative of the average child in the community	* Structured interview	** Study controls for maternal smoking during pregnancy, parental BMI, birth weight, weight gain, watching TV or play video games, sprots activities, breastfeeding, solid foods introduced before month 4, given a bottle containing milk or tea with carbohydrates to sleep, and eating snacks while watching TV	* Independent blind assessment	5
Wang et al, 2002	* Somewhat representative of the	* Structured interview	* Study controls for age	Not reported	3

	average child in the community				
Xie et al, 2007	* Somewhat representative of the average child in the community	* Structured interview	** Study controls for city residence, age, puberty, family income, parental education, parental employment and ownership of electronics	* Independent blind assessment	5

Figure S1: **Funnel plots to assess publication bias**

Plots show study size as a function of effect size for studies included in the meta-analysis. (A) Studies reporting overweight results. (B) Studies reporting obesity results. (C) Studies reporting overweight including obesity results.

A

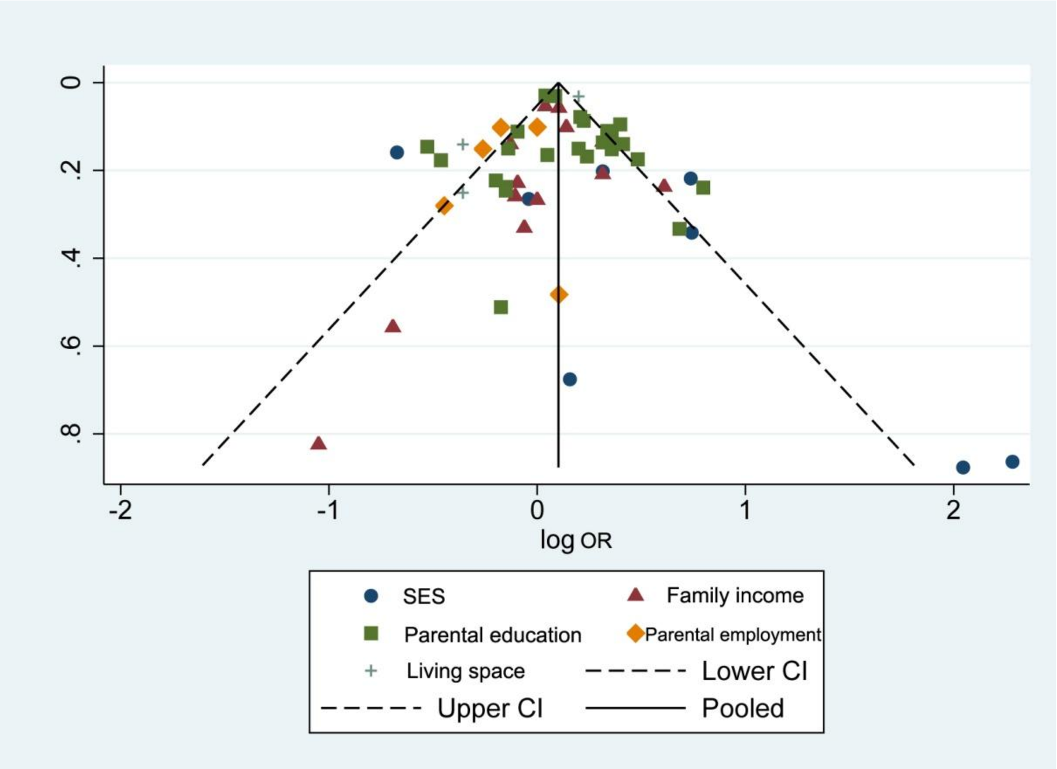

B

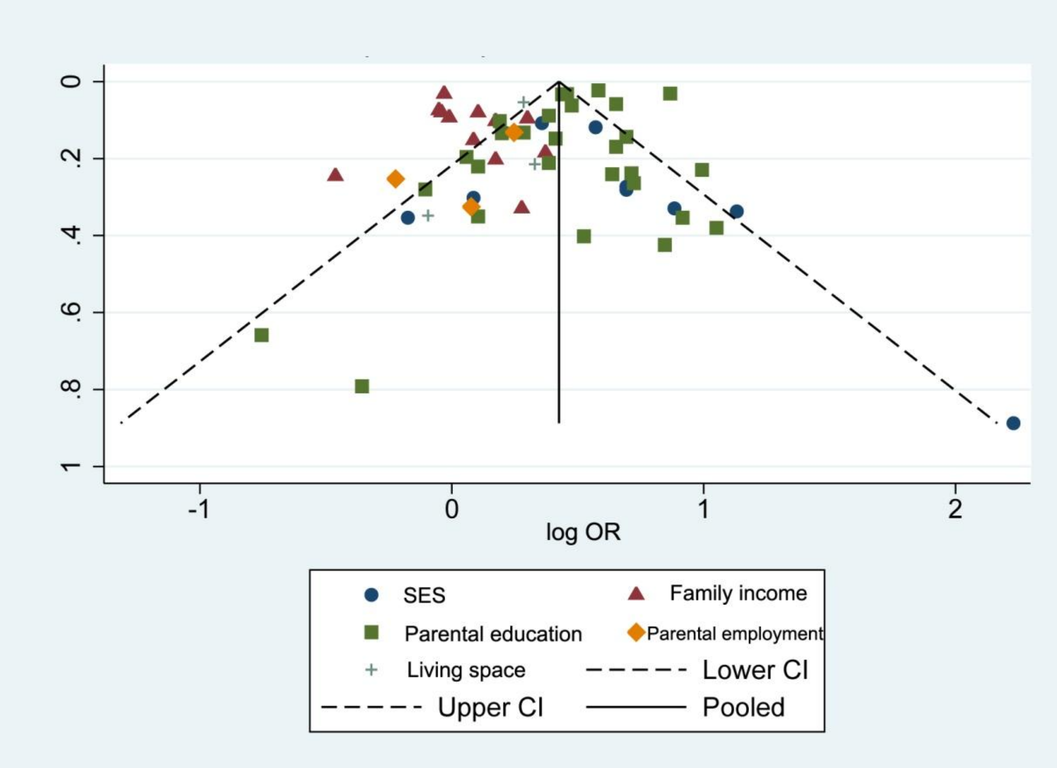

C

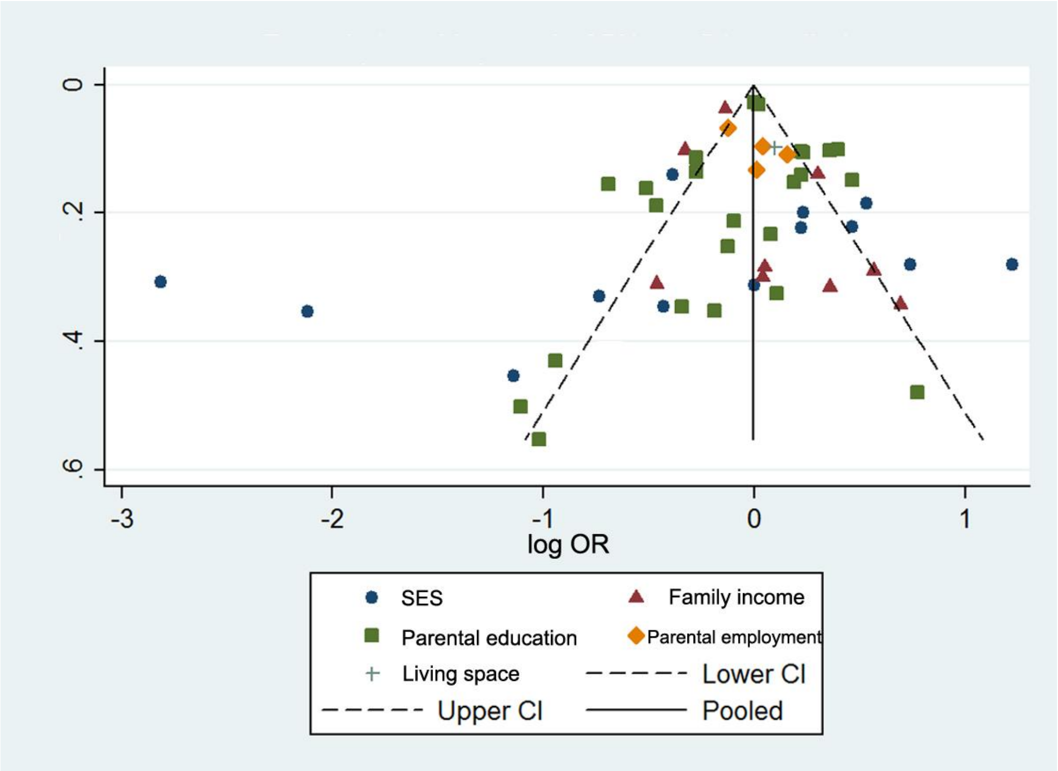
0
.2
.4
.6
-3
-2
-1
0
1
log OR
SES
Family income
Parental education
Parental employment
Living space
Lower CI
Upper CI
Pooled

Table S4: **MOOSE Checklist**

	**Criteria**	**Brief description of how the criteria were handled in the meta-analysis**
**Reporting of background should include**		
√	Problem definition	While there is a growing body of evidence that suggests that socio-economic position (SEP) is risk factor of childhood overweight and obesity, this relationship was inconsistent in different studies, and it remains to be summarized quantitatively.
√	Hypothesis statement	Family income, living space, parental educational level and car or house ownership were reported to modify children's behavior relative to energy balance, thus affecting the likelihood of childhood obesity.
√	Description of study outcomes	Childhood overweight and obesity.
√	Type of exposure or intervention used	SEP (including socio-economic status (SES), family income, parental educational level, parental employment status or living space).
√	Type of study designs used	Cohort studies and cross-sectional studies.
√	Study population	Children aged 0-15 years.
**Reporting of search strategy should include**		
√	Qualifications of searchers (eg, librarians and investigators)	The credentials of the two investigators SW and YD are provided in the author list.
√	Search strategy, including time period included in the synthesis and keywords	Medline, Web of Science, Embase, and the Cochrane Database of Systematic Reviews from 1990 to Sep 4, 2014. Keywords: "socioeconomic factors", "socio-economic", "socioeconomic", "socio economic", "wealth", "income", "education", "house size", "employment", in combination with "overweight", "obesity", "children", "childhood", "adolescent" and "teenager".
√	Effort to include all available studies, including contact with authors	References of all retrieved articles and recent reviews were reviewed.
√	Databases and registries searched	Medline, Web of Science, Embase, and the Cochrane Database of Systematic Reviews
√	Search software used, name and version, including special features used (eg, explosion)	We did not employ a special search software.
√	Use of hand searching (eg, reference lists of obtained articles)	References of all retrieved articles and recent reviews were reviewed.
√	List of citations located and	Details of the literature search process are outlined in the

	those excluded, including justification	flow chart.
√	Method of addressing articles published in languages other than English	We placed restrictions on English.
√	Method of handling abstracts and unpublished studies	The search process was not restricted upon full-text articles, but also conference abstracts and unpublished studies.
	Description of any contact with authors.	-
**Reporting of methods should include**		
√	Description of relevance or appropriateness of studies assembled for assessing the hypothesis to be tested	The inclusion criteria are presented in the “Search strategy and eligibility criteria” section.
√	Rationale for the selection and coding of data (eg, sound clinical principles or convenience)	The list of extracted data from each study pertained to the author, country, study design, sample size, age, SEP measure, outcome, number with overweight or obesity in study, risk estimates with CIs, and factors adjusted for is provided in the “Data extraction” section.
√	Documentation of how data were classified and coded (eg, multiple raters, blinding, and inrerrater reliability)	Data were independently extracted and analyzed by two investigators (SW and DY) and final decision was reached by consensus.
√	Assessment of confounding (eg, comparability of cases and controls in studies where appropriate)	Table S1 presents the adjustment factors for each study.
√	Assessment of study quality, including blinding of quality assessors; stratification or regression on possible predictiors of study results	The quality of each study was assessed by two investigators (FW and JH), using the Newcastle-Ottawa Scale.
√	Assessment of heterogeneity	The *Q*-statistic and *I*-squared (I^2) statistic were used to explore the heterogeneity among studies.
√	Description of statistical methods (eg, complete description of fixed or random effects models, justification of whether the chosen models account for predictors of study results, dose-response models, or cumulative meta-analysis) in	Description of methods of meta-analyses, subgroup analyses, and assessment of publication bias are detailed in the “Statistical analysis” section.

	sufficient detail to be replicated	
√	Provision of appropriate tables and graphics	Two main tables and three supplemental tables are provided. One flow chart and three forest plots appear in the main text.
**Reporting of results should include**		
√	Graph summarizing individual study estimates and overall estimate	Figures 2-4
√	Table giving descriptive information for each study included	Table S1
√	Results of sensitivity testing (eg, subgroup analysis)	"Results" section; Table 1
√	Indication of statistical uncertainty of findings	95% confidence intervals were presented with all summary effect estimates.
**Reporting of discussion should include**		
√	Quantitative assessment of bias (eg, publication bias)	"Results" section;<br>"Discussion" section.
√	Justification for exclusion (eg, exclusion of non-English-language citations)	The details of the exclusion of studies are shown in Flow chart.
√	Assessment of quality of included studies	Studies have been subgroup analyzed by the quality.
**Reporting of conclusions should include**		
√	Consideration of alternative explanations for observed results	We discussed that the classification of SEP may be different across studies; overweight and obesity may be defined differently according to anthropometric measurements in different countries; the measures of SEP such as parental educational level and family income may vary significantly between countries due to differences in country educational and economies systems.
√	Generalization of the conclusions (ie, appropriate for the data presented and within the domain of the literature review)	We discussed that some included studies didn't make adjustment for other factors or only make adjustment for a few important factors, thus, the risk of overweight or obesity in these studies may be contributed to other factors.
√	Guidelines for future research	We discussed that more studies are needed to quantitatively investigate the relationship of SEP and the risks of overweight and obesity in children in middle- and low-income countries. We also discussed that further studies are needed to identify the relationship of SEP and the risks of

		overweight and obesity for boys and girls separately.
√	Disclosure of funding source	The authors received no specific funding for this work.

Panel S1: **Search strategy in Medline and Embase**

1. overweight (MeSH term (Medical Subject Headings))
2. obesity (MeSH term)
3. socioeconomic factors (MeSH term)
4. socio-economic (key word)
5. socioeconomic (key word)
6. socio economic (key word)
7. wealth (key word)
8. income (key word)
9. education (key word)
10. house size (key word)
11. employment (key word)
12. survey (key word) or Data Collection (MeSH term)
13. children (key word)
14. childhood (key word)
15. adolescent (key word)
16. teenager (key word)
17. 1 or 2
18. 3 or 4 or 5 or 6 or 7 or 8 or 9 or 10 or 11 or 12
19. 13 or 14 or 15 or 16
20. 17 and 18 and 19
21. limit 20 to (English language and humans and year="1990-Current")
